# Hauterkrankungen auf nicht-weißer Haut: Rassismuskritische Lehre am Beispiel der Dermatologie

**DOI:** 10.1007/s00105-025-05544-7

**Published:** 2025-07-28

**Authors:** Marie Mikuteit, Naomi Karmann, Merve Kocyigit, Daliah Mbang Springer, Volker Paulmann, Imke von Wasielewski, Sandra Steffens

**Affiliations:** 1 Lehr- und Lernforschung, Studiendekanat Medizin, Medizinische Hochschule Hannover, Carl-Neuberg-Str. 1, 30625 Hannover, Deutschland; 2 Klinik für Dermatologie und Allergologie, Medizinische Hochschule Hannover, Hannover, Deutschland

**Keywords:** Dunklere Hauttypen, Therapieverzögerung, Hauttypen IV–VI, Diskriminierung, Soziologische Hintergründe, Darker skin types, Treatment delay, Skin types IV–VI, Racial discrimination, Cultural background

## Abstract

In der dermatologischen Lehre werden viele Erkrankungen vorrangig mit Bildern von helleren Hauttypen gelehrt. In der Praxis zeigt sich, dass Hauterkrankungen von Ärzt:innen auf dunkleren Hauttypen schlechter erkannt werden, was die Therapie verzögern kann. Aus diesem Grund wurde an der Medizinischen Hochschule Hannover ein Seminar entwickelt und durchgeführt, das sich mit Hauterkrankungen bei dunkleren Hauttypen und rassismuskritischer Lehre auseinandersetzt. Lehrende aus der Klinik für Dermatologie und der Lehrforschung entwickelten gemeinsam mit Studierenden ein 8‑stündiges Blended-Learning-Konzept für ein Seminar. Das Seminar wurde im Rahmen des Wissenschaftsmoduls angeboten. Mittels einer Prä-post-Befragung wurde der Lernzuwachs, basierend auf den Lernzielen, erfasst. Ziel des Seminars war eine Verknüpfung einer rassismuskritischen Auseinandersetzung mit medizinischen Inhalten, mit der Diagnostik von Hauterkrankungen auf dunkleren Hauttypen. Das Training der Diagnostik von Hauterkrankungen auf den Hauttypen IV–VI wurde von Hintergrundinformationen rund um die Themen rassismuskritische Lehre und Dekolonisierung eingerahmt. Das Seminar wurde 5‑mal mit insgesamt 92 Studierenden durchgeführt. In den Bereichen Dermatologie, Textarbeit und Dekolonisierung ergab sich ein signifikanter Kompetenzzuwachs. In der Domäne Diskriminierung und Reflexion gaben die Teilnehmer:innen schon vor dem Seminar hohe Werte an, dennoch zeigte sich hier ein kleiner Zuwachs. Insgesamt führte das Seminar durch die Verknüpfung von konkreten dermatologischen Fragestellungen und soziologischen Hintergründen zu signifikanten Lernzuwächsen. Zusätzlich sollte rassismuskritische Lehre longitudinal in mehreren Fächern unterrichtet werden.

**Graphic abstract:**

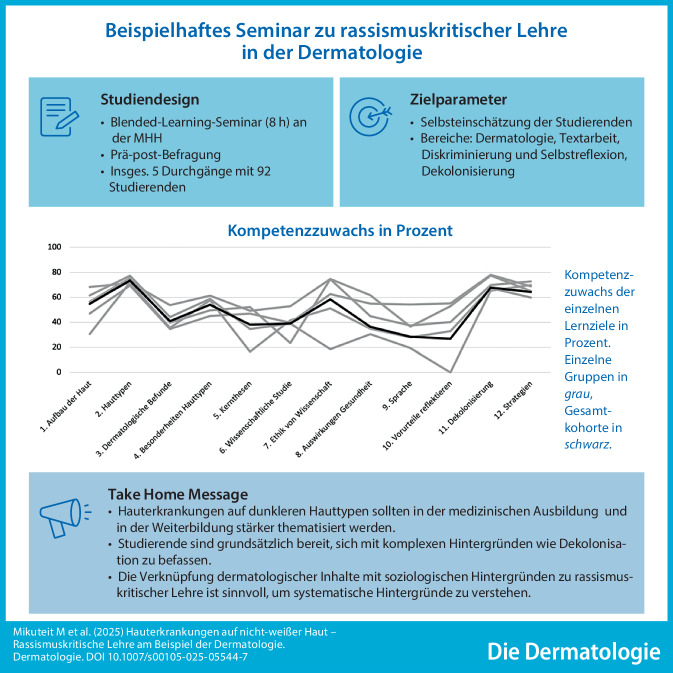

## Abstract (en)

In dermatology education, images of skin diseases are predominantly depicted on lighter skin types. Physicians are thus less likely to recognize skin diseases on darker skin types, which can delay treatment. For this reason, a seminar was developed and implemented at Hannover Medical School that addresses skin diseases on darker skin types and racism-critical teaching. Teachers from the Department of Dermatology and the Teaching Research Department developed an 8‑hour blended learning concept for a seminar in collaboration with students. The seminar was offered as part of the science module. A before-and-after survey was used to record change in competencies based on the learning objectives. The aim of the seminar was to combine a critical examination of racism in medicine with the diagnostic training of skin diseases on darker skin types. Diagnostic practice on skin types IV–VI was embedded within background content on anti-racist teaching and decolonization. The seminar was held 5 times with a total of 92 students. There was a significant increase in competence in the areas of dermatology, text work, and decolonization. In the domain of discrimination and reflection, the participants already expressed high scores before the seminar, so there was only a small increase here. Overall, the seminar led to significant learning gains by linking specific dermatological issues and sociological backgrounds. Racism-critical education should be implemented longitudinally in several subjects.

In der Dermatologie werden hauptsächlich Bilder hellerer Hauttypen zum Lernen und Lehren verwendet, sodass Ärzt:innen Hauterkrankungen auf dunkleren Hauttypen schlechter erkennen. Im Nationalen Kompetenzbasierten Lernzielkatalog Medizin sind Lernziele zu rassismuskritischer Lehre hinterlegt. Dennoch werden Rassismus und dessen Folgen für die Gesundheit in der medizinischen Ausbildung in Deutschland nicht ausreichend behandelt. An der Medizinischen Hochschule Hannover wurde ein Seminar über Rassismus in der Medizin eingeführt, welches das Thema am Beispiel der Dermatologie beleuchtet.

## Hintergrund und Fragstellung

Im Fach Dermatologie spielen das Erkennen und Befunden von Hauterkrankungen per Blickdiagnose und somit Bilder als Lehrmaterial eine besondere Rolle. Dennoch werden in diesem Fach hauptsächlich Abbildungen hellerer Hauttypen verwendet [1]. In deutschsprachigen Lehrbüchern lassen sich demnach 91,4 % der Abbildungen den Hauttypen I oder II nach Fitzpatrick zuordnen, wohingegen die Hauttypen V und VI nur von 1,41 % der Abbildungen repräsentiert werden [2]. Wenn wir im Folgenden von „dunklerer Haut“ sprechen, beziehen wir uns auf die Fitzpatrick-Hauttypen IV–VI, die in der dermatologischen Lehre deutlich unterrepräsentiert sind. Mit einem Schwerpunktheft hat *Die Dermatologie* 2023 einen Fokus auf Dermatosen auf „skin of color“ gelegt mit dem Ziel, die fachliche Expertise gezielt zu unterstützen [3].

Darüber hinaus bleibt durch eine fehlende Verankerung der Diagnostik auf allen Hauttypen – sowohl im Nationalen Kompetenzbasierten Lernzielkatalog Medizin (NKLM) als auch in der Weiterbildungsordnung für Haut- und Geschlechtskrankheiten – eine Integration von bestehendem Bildmaterial in die medizinische Ausbildung aus. Die Studierenden diagnostizieren Hauterkrankungen auf dunklerer Haut signifikant schlechter als auf hellerer Haut [4]. Auch Dermatolog:innen fühlen sich aktuell nicht ausreichend darin ausgebildet, Hauterkrankungen wie Hyperpigmentierung auf dunkleren Hauttypen zu diagnostizieren [5]. Konkrete Konsequenzen dessen zeigen sich unter anderem in einer 2‑ bis 3fach erhöhten Mortalität von Patient:innen mit malignem Melanom mit dunklerer Haut gegenüber Patient:innen mit hellen Hauttypen [6].

Darüber hinaus erfahren nicht-weiße Patient:innen auch im ärztlichen und dermatologischen Behandlungssetting rassistische Diskriminierung, die sich wiederum negativ auf ihre Gesundheit auswirken kann [7]. Auch Medizinstudierende nehmen Rassismus als allgegenwärtiges Problem wahr und äußern sowohl im Erkennen von rassistischen Strukturen und Verhaltensweisen als auch im Umgang mit rassistischen Situationen Unsicherheiten [8]. Vor diesem Hintergrund haben sich Lehrende und Studierende an der Medizinischen Hochschule Hannover (MHH) die Frage gestellt, wie ein rassismuskritischer Zugang in der Lehre gelingen kann, der exemplarisch die Perspektive der Dermatologie vertieft.

Als Ansatzpunkt für die Erweiterung der fehlenden fachspezifischen Ausbildungsinhalte in der Dermatologie sollten deshalb auch theoretische Auseinandersetzungen mit rassistischen Narrativen und Strukturen sowie mit den historischen Zusammenhängen von Kolonialismus und Medizin in das medizinische Curriculum der MHH integriert werden. In Anlehnung an postkoloniale Theorieansätze verstehen wir unter „Dekolonisierung in der Medizin“ im Kontext unseres Seminars die kritische Auseinandersetzung und Überwindung historisch gewachsener, kolonial geprägter Strukturen und Denkmuster im medizinischen Kontext. Dies umfasst die Anerkennung und aktive Adressierung von Repräsentationslücken, wie sie beispielsweise bei der unzureichenden Abbildung verschiedener Hauttypen in dermatologischen Lehrmaterialien deutlich werden [9].

Damit sollen die Studierenden in die Lage versetzt werden, sowohl gesellschaftliche als auch individuelle Verhaltensmuster zu erkennen. Im besten Fall kann dies zu einer verbesserten Behandlung aller Patient:innen führen – unabhängig vom konkreten fachlichen Kontext. Dieses übergeordnete Lernziel wurde auch in den NKLM aufgenommen (VIII.6–04.4.13; https://www.nklm.de).

Im Folgenden werden die anvisierten Lernziele, die Konzeption sowie die konkrete Umsetzung eines Seminars zum Thema „Was hat Dermatologie mit Rassismus zu tun?“ vorgestellt. Auf der Basis von 5 Durchläufen werden zudem erste Ergebnisse der durchgeführten Lernzuwachsmessung im Kontext der Lehr- und Lernforschung beleuchtet, die die weitere Auseinandersetzung um eine zeitgemäße dermatologische Ausbildung anregen sollen.

## Studiendesign und Untersuchungsmethoden

### Konzeption des Seminars

Das Wahlpflicht-Seminar „Was hat Dermatologie mit Rassismus zu tun?“ wurde im Rahmen des Wissenschaftsmoduls konzipiert und in Zusammenarbeit mit der Klinik für Dermatologie der MHH, studentischen Hilfskräften und dem Bereich Lehr- und Lernforschung des Studiendekanats der MHH entwickelt [10]. Die Konzeption erfolgte von November 2021 bis März 2022 und folgte der Curriculumentwicklung nach Kern [11]. Lernziele wurden anhand des NKLM 2.0 sowie einer Publikation von Finke et al. [12] ausgewählt und sprachlich adaptiert. Das Seminar umfasst insgesamt 8 h, davon verteilen sich 4 h auf 2 Präsenzsitzungen, die verbleibenden 4 h werden als asynchrone Lehre von Studierenden selbstständig online im Lernmanagementsystem ILIAS und in Kleingruppen erarbeitet. Das erarbeitete Material wurde von Studierenden getestet und im Hinblick auf Verständlichkeit der Aufgaben und benötigte Zeit evaluiert. Mit dieser Rückmeldung wurde insbesondere der asynchrone Seminarteil überarbeitet und angepasst.

### Durchführung des Seminars

Pro Veranstaltung konnten maximal 20 Studierende aus dem 2. bis 4. Studienjahr teilnehmen. Die Studierenden hatten zu Beginn der Veranstaltung im Rahmen des Wissenschaftsmoduls bereits mehrere Assessment-Portfolio-Aufgaben bearbeitet, in denen sie unter anderem ihr eigenes Verständnis von Wissenschaft erläutert, reflektiert und erweitert und Grundlagen der Literaturrecherche erlernt hatten, unter anderem eine eLearning-Aufgabe zum Thema Rassismus in der Medizin [10]. Die Studierenden konnten die Teilnahme am Seminar im Wahlpflichtmodus wählen.

### Qualitätssicherung und Wissenszuwachs

Um den studentischen Wissenszuwachs abbilden zu können, wurde in Anlehnung an Raupach et al. [13] zu 2 Erfassungs- und Beobachtungszeitpunkten (jeweils vor und nach dem Seminar) eine Befragung durchgeführt. Dabei sollten die Teilnehmer:innen ihre Fähigkeiten mit Blick auf die im Vorfeld festgelegten Lernziele einschätzen. Durch diese Selbsteinschätzung der praktischen, kognitiven und affektiven Kompetenzen kann für die Studierenden ein prozentualer Lernzuwachs (nach Raupach) ermittelt werden. Die Fragen konnten auf einer siebenstufigen Likert-Skala von 1 („trifft gar nicht zu“) bis 7 („trifft voll zu“) beantwortet werden. Die Umfrage wurde für die ersten beiden Durchgänge anonym durchgeführt, sodass eine direkte Zuordnung der Vorher- und Nachher-Werte nicht möglich war. Für die anschließenden Durchgänge 3 bis 5 wurde eine pseudonymisierte Umfrage durchgeführt, die eine direkte Zuordnung der Vorher- und Nachher-Werte erlaubt. Die Auswertung erfolgte in Microsoft Excel. Für die einzelnen Fragen wurden Mittelwerte (MW) und Standardabweichung (SD) sowie die Differenz der MW und die prozentuale Veränderung der Kompetenz nach Raupach et al. berechnet [13].

Die Null-Hypothese lautete, dass es keinen Lernzuwachs bei den Studierenden im Prä-post-Vergleich gab. Verglichen wurden die Mittelwerte der Items mittels (gepaarten) t‑Tests für alle Durchgänge. Ein *p*-Wert < 0,05 wurde als signifikant betrachtet. Die *p*-Werte der Vergleiche des Kompetenzzuwachses wurden mittels Bonferroni-Holm Methode für multiples Testen korrigiert.

Zusätzlich konnten die Studierenden offenes Feedback und Vorschläge anbringen.

## Ergebnisse

### Konzeption des Seminars

Als übergeordnetes Lernziel des Seminars stand die Verknüpfung von konkreten Beispielen der Dermatologie mit gesellschaftspolitischen Hintergründen im Vordergrund. Neben der Vermittlung von Kompetenzen bei der Diagnostik von Hautveränderungen auf dunkleren Hauttypen (Fitzpatrick-Hauttypen IV–VI) erfolgte eine Auseinandersetzung mit kolonialen und rassistischen Strukturen innerhalb der Medizin auf mögliche Ursprünge für die existierende Ungleichbehandlung. Die einzelnen Lernziele bezogen sich auf inhaltliche (I) und methodische (M) Kompetenzen sowie auf Kompetenzen der Reflexions- (R) und Kritikfähigkeit (K) (Tab. 1). Die inhaltlichen Kompetenzen wurden weiter unterteilt in die Themenbereiche Dermatologie (Der), Stigmatisierung und Diskriminierung (StDi) und Dekolonisierung (Dek).

**Tab. 1:** Lernziele des Seminars „Was hat Dermatologie mit Rassismus zu tun?“ mit NKLM-Verweisen

Kompetenzbereich	Lernziel: Die Studierenden können …		NKLM-Lernziele
Dermatologie	1.1	… den mikroanatomischen Aufbau der Haut sowie mikroanatomische Grundlagen verschiedener Hauttypen erklären	–
	1.2	… nationale und internationale dermatologische Klassifikationssysteme bezüglich Hauttypen inklusive ihrer Vor- und Nachteile in der Anwendung benennen	V.II‑2.01.1.3
	1.3	… Beispiele für physiologische und pathologische Besonderheiten dunklerer Hauttypen nennen	–
	1.4	… den diagnostischen Prozess als Prozess additiven und/oder linearen Schlussfolgerns verstehen und kritisch bewerten	VII.2-01.1.1
	1.5	… alle Hauttypen auf gleichem Kompetenzniveau beurteilen, sind sich der unterschiedlichen Präsentation auf hellen und dunklen Hauttypen bewusst und können diagnostische Verfahren entsprechend auswählen und anpassen	–
Stigmatisierung und Diskriminierung	1.6	… Gesellschaftliche Stigmatisierungsprozesse in ihren Auswirkungen auf Gesundheit und Krankheit und Behinderung berücksichtigen	VIII.2-05.2.2
	1.7	… Diversitätsbezogene Aspekte der Patient:innen berücksichtigen	VIII.6-04.1.2
	1.8	… Unterschiedliche Konzepte von Gerechtigkeit und ihre Konsequenzen für die Medizin erläutern	VIII.6-02.3.1
	1.9	… Benachteiligungen, Stigmatisierung und Diskriminierung aus rassistischen Gründen erkennen und das Handeln im Sinne der Verhinderung oder Beseitigung dieser Benachteiligungen ausrichten	VIII.6-04.4.13
Dekolonisierung	1.10	… den Begriff „Dekolonisierung“ erklären	–
	1.11	… Strategien benennen, wie sich Institutionen im Gesundheitswesen im Sinne einer Gleichbehandlung aller Menschen verändern ließen	–
Methodik	2.1	… die Kernthesen eines Textes herausarbeiten und formulieren	–
	2.2	… Versuchsaufbauten und Forschungsergebnisse anhand der aktuell geltenden ethischen Normen einordnen; dafür wissenschaftliches Wissen von anderen Wissensformen unterscheiden und ethische Konflikte erkennen, diese analysieren und damit in der Praxis professionell umgehen	VIII.1-01.2.1 VIII.6-01.2.9
	2.3	… historische Entwicklungen des Gesundheitssystems und deren ethisch relevante Unterschiede erläutern	VIII.6-02.2.1
	2.4	… relevante (Sekundär- und Tertiär‑)Literatur und andere Informationsquellen mit geeigneten Recherchesystemen und effektiven Suchstrategien recherchieren, eine Auswahl treffen und interpretieren	VIII.1-02.1.4
	2.5	… wissenschaftliches Wissen von anderen Wissensformen unterscheiden, z. B. historisch geprägtes „Pseudowissen“ mit diskriminierenden Auswirkungen wie rassistische Falschannahmen	–
	2.6	… medizinische Verbrechen zu Zeiten des Kolonialismus, der Sklaverei und Apartheid erläutern. Sie kennen die Rolle der Medizin in der Konstruktion und Legitimierung von Rassetheorien sowie deren Bedeutung im Kontext von rassistischen Verbrechen. Sie setzen sich kritisch mit der Aufrechterhaltung von kolonialen und rassistischen Denkmustern in der Medizin bis heute auseinander. Sie sind sich der bisher eingeschränkten gesellschaftlichen Aufarbeitung bewusst	–
Reflexion	3.1	… die eigene moralische Position reflektieren, weiterentwickeln und argumentativ vertreten	VIII.6-03.1.7
	3.2	… sich selbst und ihr Handeln beobachten und kritisch reflektieren	VIII.6-03.1.1
	3.3	… eigene Vorurteile und Stereotype benennen und reflektieren	–
	3.4	… ihre eigene künftige Rolle als Ärzt:in in der Gesellschaft einschätzen, reflektieren und entwickeln	VIII.5-01.1
	3.5	… eine gesteigerte Aufmerksamkeit entwickeln, um mit größtmöglicher Achtsamkeit und Präsenz den Patient:innen und ihren Belangen zu begegnen	VIII.5-11.1.11
Kritik	4.1	… sachliche Kritik üben sowie solche annehmen, reflektieren und gegebenenfalls ihr Verhalten verändern	VIII.6-03.1

*NKLM* Nationaler Kompetenzbasierter Lernzielkatalog Medizin

Um einen möglichst breiten Zugang zu ermöglichen und verschiedene Perspektiven zu fördern, wurden Studierende aus dem 2. bis 4. Studienjahr adressiert.

**Abb. 1:**
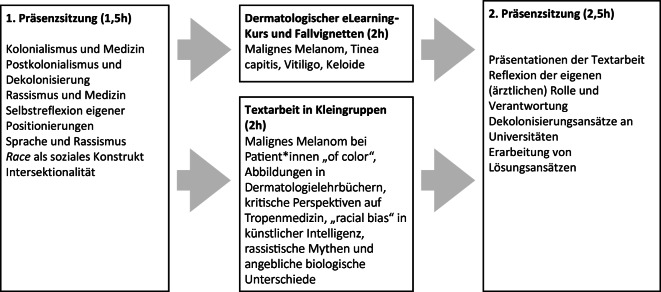
Aufbau des Seminars „Was hat Dermatologie mit Rassismus zu tun?“. Schematische Übersicht der Seminarinhalte und des -aufbaus

Das Seminar umfasste insgesamt 8 h im Blended-Learning-Format (Abb. 1). Die erste Präsenzsitzung (1,5 h) dient dem Kennenlernen der Gruppe und einer allgemeinen Einführung in das Themenfeld der rassismuskritischen Medizin. Danach wurden nach einem allgemeinen Input zum Aufbau der Haut aus dem Institut für Anatomie der MHH und einer allgemeinen Effloreszenzenlehre 4 dermatologische Fallvignetten, die gezielt die diagnostischen Besonderheiten und Herausforderungen bei Hauttypen IV–VI darstellen, asynchron und digital von den Studierenden einzeln bearbeitet. Zu den Themen Tinea corporis, Vitiligo, akrolentiginöses Melanom und Keloide wurden jeweils fiktive Patient:innen vorgestellt, und die Studierenden beantworteten Fragen bezüglich der Effloreszenz, weiterer Untersuchungsschritte und der wahrscheinlichsten Diagnose. Das Bildmaterial stammte aus der Bilddatenbank der MHH und aus der Public Health Image Library des CDC (Cancer Prevention and Control). Parallel vertieften sie durch Textarbeit in Kleingruppen von 3 bis 4 Studierenden die Verknüpfung zwischen klinischen und gesellschaftlichen Aspekten (je 2 h pro Gruppe): zum malignen Melanom bei Patient:innen „of color“, zum Stand der Abbildungen in Dermatologielehrbüchern, zu kritischen Perspektiven auf die historischen Ursprünge der Tropenmedizin, zum „racial bias“ in künstlicher Intelligenz oder zu immer noch existierenden rassistischen Mythen hinsichtlich vermeintlicher biologischer Unterschiede. In der zweiten Präsenzsitzung (2,5 h) stellten die Gruppen ihre Ergebnisse vor, gefolgt von einer kurzen moderierten Diskussion pro Thema (ca. 10 min je Text). Dieser Prozess förderte nicht nur den inhaltlichen Austausch, sondern machte auch die konkreten Verbindungen zwischen den dermatologischen Erkrankungen und den strukturellen Dimensionen der Dekolonisierung sichtbar. Zusätzlich konnten auch die Präsentationskompetenzen der Studierenden geschult werden. Eine anschließende Diskussions- und Reflexionsrunde sollte den Transfer der erarbeiteten Themen in das eigene studentische und ärztliche Handeln begleiten. Nach einem Kurzvortrag zum Thema „Dekolonisierung“ wurden gemeinsam Ansätze für antirassistische Maßnahmen an der eigenen Universität gesammelt. Die Evaluation der Studierenden vor der Pilotierung führte zu einer Überarbeitung insbesondere des asynchronen Seminarteils.

### Durchführung des Seminars

Das Seminar wurde zwischen April 2022 und November 2023 insgesamt 5‑mal durchgeführt, insgesamt nahmen 92 Studierende aus dem 2. (11 %), 3. (88 %) und 4. (1 %) Studienjahr teil (Tab. 2). Das durchschnittliche Alter betrug 24,2 (SD 2,8) Jahre; 79 % der Teilnehmenden gaben ihr Geschlecht als weiblich an (Tab. 2). Das Modul Dermatologie findet im 4. Studienjahr statt, sodass die meisten Studierenden dieses Modul noch nicht absolviert hatten. Die Studierenden bewerteten die investierte Zeit und den Lernzuwachs in direkten Rückmeldungen als ausgeglichen. Von einigen Studierenden wurden mehr dermatologische Inhalte gewünscht, sodass ein digitaler Input zu dermatologischen Grundlagen (dermatologische Klassifikationssysteme, diagnostische Grundlagen in der Dermatologie und physiologische und pathologische Besonderheiten verschiedener Hauttypen) ausgeweitet und als vertonte Vorlesung (ca. 35 min) bereitgestellt wurde.

**Tab. 2:** Demografische Daten der Seminarteilnehmer:innen

		1. Durchgang	2. Durchgang	3. Durchgang	4. Durchgang	5. Durchgang	Gesamt
Anzahl		20	20	20	14	18	92
Gender, *n* (%)	Weiblich	17 (85)	15 (75)	18 (90)	11 (79)	12 (67)	73 (79)
	Männlich	3 (15)	4 (20)	1 (5)	3 (21)	6 (33)	17 (19)
	Divers	–	–	–	–	–	0 (0)
	k. A.	–	1 (5)	1 (5)	–	–	2 (2)
Alter (Jahre), MW (SD)		23,9 (2,1)	24,8 (3,3)	23,7 (2,6)	24,9 (3,8)	23,6 (2,1)	24,2 (2,8)
Studienjahr, *n* (%)	2	–	–	–	10 (71)	–	10 (11)
	3	20 (100)	20 (100)	20 (100)	4 (29)	17 (94)	81 (88)
	4	–	–	–	–	1 (6)	1 (1)

*N* Anzahl, *k.* *A.* keine Angabe, *MW* Mittelwert, *SD* Standardabweichung

### Qualitätssicherung und Wissenszuwachs

**Abb. 2:**
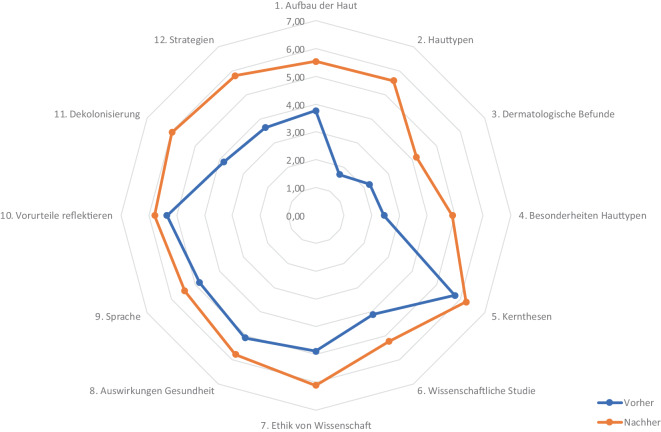
Selbsteinschätzung der Studierenden vor und nach dem Seminar. Dargestellt sind die Mittelwerte der Selbsteinschätzung vor (*blau*) und nach (*orange*) dem Seminar

Bei der Selbsteinschätzung der Studierenden zeigten sich niedrigere Werte in der Prä-Testung in den Kompetenzen der Bereiche Dermatologie und Dekolonisierung (zwischen 1,7 und 3,83 Punkten). Kompetenzen bezüglich Textarbeit und Diskriminierung und Selbstreflexion bewerteten die Studierenden als eher vorhanden (4,11 bis 5,76 Punkte). Für alle Fragen zeigte sich ein signifikanter Unterschied zwischen den Prä- und Post-Werten mit Differenzen zwischen 0,44 und 3,9 Punkten bzw. 26,9 und 73,5 % (Tab. 3 und Abb. 2).

**Tab. 3:** Vergleich der studentischen Selbsteinschätzung der Kompetenzen (Lickert-Skala 1–7) vor und nach dem Seminar

		Prä, MW (SD)	Post, MW (SD)	Differenz	Zuwachs in %	*p*-Wert*
Dermatologie	1. Ich kann den mikroanatomischen Aufbau der Haut sowie mikroanatomische Grundlagen verschiedener Hauttypen erklären	3,76 (1,27)	5,53 (0,95)	1,77	54,7	< 0,001
	2. Ich kann internationale dermatologische Klassifikationssysteme bezüglich Hauttypen inklusive ihrer Vor- und Nachteile in der Anwendung benennen	1,70 (1,06)	5,59 (1,00)	3,90	73,5	< 0,001
	3. Ich kann dermatologische Befunde korrekt beschreiben	2,23 (1,21)	4,17 (1,13)	1,95	40,8	< 0,001
	4. Ich kann Beispiele für physiologische und pathologische Besonderheiten von hellen Hauttypen gegenüber dunkleren Hauttypen nennen	2,45 (1,46)	4,91 (0,99)	2,46	54,1	< 0,001
Textarbeit	5. Ich kann die Kernthesen eines Textes herausarbeiten und formulieren	5,76 (0,80)	6,23 (0,88)	0,47	38,2	< 0,001
	6. Ich kann eine wissenschaftliche Studie, ihre Methodik und ihre Forschungsergebnisse anhand der aktuell geltenden ethischen Normen beurteilen	4,11 (1,30)	5,23 (1,30)	1,12	39,0	< 0,001
	7. Ich kann historische Beispiele für unethische medizinische Versuche nennen	4,88 (1,75)	6,12 (0,98)	1,24	58,4	< 0,001
Diskriminierung und Selbstreflexion	8. Ich kann anhand konkreter Beispiele die Auswirkungen von sozialer Ungleichheit und Diskriminierung auf Gesundheit und Krankheit erläutern	5,09 (1,30)	5,78 (1,34)	0,69	36,3	0,041
	9. Ich kann diskriminierungssensible Sprache sicher anwenden	4,83 (1,21)	5,44 (1,28)	0,62	28,4	0,041
	10. Ich kann eigene Vorurteile und Stereotype benennen und reflektieren	5,35 (1,03)	5,79 (1,32)	0,44	26,9	0,041
Dekolonisierung	11. Ich kann den Begriff Dekolonisierung erklären	3,83 (1,67)	5,98 (1,20)	2,15	67,8	< 0,001
	12. Ich kann Strategien benennen, wie sich Institutionen im Gesundheitswesen im Sinne einer Gleichbehandlung aller Menschen verändern ließen	3,64 (1,40)	5,80 (1,15)	2,16	64,4	< 0,001

*MW* Mittelwert, *SD* Standardabweichung

* Vergleich der Stichproben mittels gepaarten t‑Tests, *p*-Werte mittels Bonferroni-Holm-Methode korrigiert. Kompetenzzuwachs in Prozent nach Raupach et al. [13]

In der strukturierten Analyse der mündlichen Rückmeldungen kristallisierten sich 2 Hauptkategorien heraus. Die Teilnehmenden beschrieben einen signifikanten Wissenszuwachs beim Verständnis der direkten Auswirkungen mangelnder Repräsentation auf die diagnostische Kompetenz. Außerdem wurde die praktische Relevanz der vermittelten Inhalte für die zukünftige klinische Tätigkeit betont, aber auch im Hinblick auf rassismuskritische Lehre in anderen Fächern.

## Diskussion

Das Seminar „Was hat Dermatologie mit Rassismus zu tun?“ ist die erste Lerneinheit an der MHH, in der das Thema rassistische Diskriminierung in der Medizin als gesellschaftliches Problem vor dem Hintergrund konkreter klinischer Beispiele diskutiert wird. Die Verknüpfung von theoretischen und historischen Perspektiven am Beispiel des Konzeptes der „Dekolonisierung“ mit praktischen Beispielen von dermatologischen Erkrankungen zielt darauf ab, das Verständnis der komplexen Zusammenhänge zwischen Rassismus und Gesundheit zu verbessern. Eine besondere Herausforderung in der medizinischen Lehre besteht darin, den Begriff „Race“ als Analysekategorie kritisch zu reflektieren und ihn von dem historisch belasteten deutschen Begriff „Rasse“ abzugrenzen, während gleichzeitig die realen gesundheitlichen Auswirkungen von struktureller Diskriminierung anerkannt werden [8].

Eine flächendeckende praktische Umsetzung rassismuskritischer Positionen in die Curricula der deutschen medizinischen Hochschulen steht noch aus. Dennoch verdeutlichen Initiativen der GMA (Gesellschaft für Medizinische Ausbildung) oder bvmd (Bundesvertretung der Medizinstudierenden in Deutschland e. V.) [12, 14], dass der inzwischen breit geführte gesellschaftliche Diskurs auch von Ärzt:innen mitgestaltet wird. Die soziale Verantwortung ist nicht nur mit Blick auf die Patient:innenversorgung geboten, sondern auch im Alltag der Klinik oder im Studium.

Das vorgestellte Seminar stellt einen niedrigschwelligen Lehrimpuls im dicht gedrängten Medizinstudium dar. Als Wahlpflicht-Seminar können bestehende Strukturen genutzt werden, anstatt zusätzliche Zeitfenster zu fordern. Durch das Blended-Learning-Format können die Studierenden Inhalte im eigenen Tempo bearbeiten und ggf. mehrfach wiederholen. Zudem ist eine flexible Integration der asynchronen Lehreinheiten in zeitintensive Stundenpläne möglich [15]. Der Austausch in den studentischen Arbeitsgruppen führt zu einer multidimensionalen Betrachtung, und zugleich werden Präsentationskompetenzen geschult. Die Mitarbeit von Studierenden an der Entwicklung der Inhalte erwies sich ebenfalls als effektiv, da das Seminar so auf die konkreten Bedürfnisse der Peergruppe abgestimmt werden konnte [16].

Die Rückmeldungen der Teilnehmer:innen zeigen, dass das Seminar im Wissenschaftsmodul gut angenommen wurde. Die angebotenen Kursplätze wurden nachgefragt. Durch das nach der ersten Evaluation erweiterte Lehrmaterial zu den Grundlagen der Dermatologie waren die Studierenden nach eigener Aussage auch besser in der Lage, die dermatologischen Fälle zu bearbeiten. Ebenso wie in anderen Untersuchungen konnten wir zeigen, dass schon kleine Interventionen zugunsten der Sichtbarkeit von dunkleren Hauttypen im Lehrkontext von Studierenden positiv bewertet werden [17]. Basierend auf den Ergebnissen des Pilotprojekts, wurde im regulären Dermatologiemodul ein Implementierungsprozess gestartet, der zunehmend Bildmaterial zu dunkleren Hauttypen integriert. Diese Erweiterung wird didaktisch begleitet, um das diagnostische Bewusstsein für unterschiedliche Hauttypen zu stärken.

Die Selbsteinschätzung der Studierenden ergab einen signifikanten Kompetenzzuwachs in den Bereichen der „Dermatologie“, der „Textarbeit“ und Wissen über „Dekolonisierung“. Ein ebensolcher Lernzuwachs konnte auch in anderen Studien beobachtet werden [18]. Es muss kritisch angemerkt werden, dass die meisten teilnehmenden Studierenden das reguläre Dermatologiemodul im Curriculum noch nicht absolviert hatten. Diese Tatsache erklärt die niedrigen Ausgangswerte in der Selbsteinschätzung der dermatologischen Kompetenzen und könnte den erheblichen subjektiven Wissenszuwachs in diesem Bereich teilweise erklären. Der Neuheitseffekt dermatologischer Lehrinhalte stellt somit einen methodischen Limitationsfaktor bei der Interpretation der Ergebnisse dar. Gleichwohl wurde dieser Umstand bei der Konzeption des Seminars berücksichtigt, indem auf den Lernstand der Studierenden abgestimmte Materialien entwickelt wurden, die einen niedrigschwelligen Einstieg in die dermatologischen Grundlagen ermöglichten.

Besonders bemerkenswert erscheint vor diesem Hintergrund der ebenfalls signifikante Wissenszuwachs im Bereich „Dekolonisierung“, was auf die erfolgreiche Verknüpfung klinischer und gesellschaftskritischer Inhalte hindeutet. Im Bereich Diskriminierung und Selbstreflexion gaben die Studierenden bereits ein relativ hohes Ausgangsniveau ihrer Kompetenzen an, was möglicherweise auf die bereits absolvierte eLearning-Aufgabe zum Thema „Rassismus in der Medizin“ zurückgeführt werden kann. Auch kann hier eine Positivauswahl bei den Teilnehmer:innen vorliegen: Der thematische Fokus des Seminars könnte insbesondere Studierende angesprochen haben, die an der sozialen und historischen Einbettung des Themas besonders interessiert waren. Dessen ungeachtet gaben die Studierenden das Vorwissen in den Bereichen Dermatologie und „Dekolonisierung“ insgesamt als eher niedrig an, was für einen Bedarf an Lehrinhalten spricht, die sich mit dem Konzept von Rassismus und Dekolonisierung in der Medizin befassen [8].

Das Seminar „Was hat Dermatologie mit Rassismus zu tun?“ bietet einen Einstieg in das komplexe Thema von Rassismus im Gesundheitswesen anhand eines ausgewählten klinischen Kontextes. Statt fachspezifische und gesellschaftliche Inhalte als separate Themen zu behandeln, wurden sie hier integrativ verknüpft. Insgesamt sollten Impulse zum Thema Rassismus in der Medizin longitudinal in das bestehende Curriculum eingewoben werden, um Dozierende und Studierende gleichermaßen weiterzubilden und den Diskurs zu stärken. Dies wurde bereits von Medizinstudierenden 2023 gefordert [8, 12]. Bezogen auf die Dermatologie können beispielsweise vermehrt Bilder von Hauterkrankungen auf unterschiedlichen Hauttypen in die Lehre eingebunden werden, um einer Ungleichbehandlung vorzubeugen.

Insgesamt gibt es bislang – im Vergleich zu englischsprachigen Lernangeboten (z. B. https://www.medicalantiracismcurriculum.com) – noch wenige Methodenbeschreibungen und offene Bildungsmaterialien in deutscher Sprache. Aus diesem Grund wurden das Konzept dieses Seminars sowie die erstellten Materialien als Open Educational Ressource veröffentlicht (www.twillo.de), um damit Anreize zur Durchführung an anderen Fakultäten zu geben.

Eine Limitation unserer Evaluation liegt in der ausschließlichen Nutzung von Selbsteinschätzungen, die durch soziale Erwünschtheit verzerrt sein können. Künftig wäre die Ergänzung durch objektive Tests oder Follow-up-Erhebungen zur Nachhaltigkeit des Gelernten sinnvoll. Dennoch zeigen die durchgehend positiven Rückmeldungen, dass die Verbindung von dermatologischer Praxis und Reflexion bei den Studierenden gut angenommen wird und selbstreflektiertes Lernen fördert.

## Fazit für die Praxis

- Hauterkrankungen auf nicht-weißer Haut sollten sowohl in der medizinischen Ausbildung als auch in der Weiterbildung stärker thematisiert werden.
- Studierende sind grundsätzlich bereit, sich mit theoretischen gesellschaftlichen Konzepten wie Dekolonisierung auseinanderzusetzen.
- Die Verknüpfung dermatologischer Inhalte mit historischen und soziologischen Hintergründen erscheint sinnvoll, um systematische Perspektiven auf eine rassismuskritische Lehre zu entwickeln.
- Lehrmaterial ist verfügbar über twillo: Naomi Karmann, Marie Mikuteit, Daliah Mbang Springer, Merve Kocyigit, Imke von Wasielewski, Sandra Steffens, Volker Paulmann (2024): Was hat Dermatologie mit Rassismus zu tun? Lehrkonzept für ein Seminar im Rahmen des Wissenschaftsmoduls für das 3. und 4. Studienjahr an der Medizinischen Hochschule Hannover (MHH). Open Educational Ressources (OER): CC-BY (4.0). https://www.twillo.de/edu-sharing/components/render/d1f6c6dc-eb41-4686-b2ec-9292e526278d/.

## Data Availability

Die in dieser Studie erhobenen Datensätze können auf begründete Anfrage beim Korrespondenzautor angefordert werden.
